# Inelastic Behavior of Polyoxymethylene for Wide Strain Rate and Temperature Ranges: Constitutive Modeling and Identification ^†^

**DOI:** 10.3390/ma14133667

**Published:** 2021-07-01

**Authors:** Yevgeniya Filanova, Johannes Hauptmann, Frank Längler, Konstantin Naumenko

**Affiliations:** 1 THK RHYTHM AUTOMOTIVE GmbH, D-40233 Duesseldorf, Germany; yevgeniya.filanova@ovgu.de (Y.F.); Johannes.Hauptmann@trde.thk.com (J.H.); frank.laengler@trde.thk.com (F.L.); 2 Institute of Mechanics, Otto-von-Guericke-University Magdeburg, D-39106 Magdeburg, Germany

**Keywords:** polyoxymethylene, rate-dependent inelasticity, composite model, inelastic dilatation

## Abstract

The aim of this paper is to present experimental data and the constitutive model for the inelastic behavior of polyoxymethylene in wide strain rate and temperature ranges. To capture the non-linearity of the stress responses for both loading and unloading regimes, the composite model of inelastic deformation is utilized and further developed. The equivalent inelastic strain rate is described by the Prandtl–Eyring law, while the temperature dependence is characterized by the modified Arrhenius-type law. Generalized equivalent stress and the flow rule are formulated to capture pressure sensitivity, transverse strain and volumetric strain responses. The results obtained by the constitutive law are compared with experimental data for stress vs. axial strain from standard tension tests as well as with axial and transverse strains measured by digital image correlation. The developed composite model is able to capture the non-linearity of stress–strain curves for complex loading paths within the small strain regime. For higher strains, apart from geometrically non-linear theory, evolution laws for the volume fraction of the constituents should be modified and calibrated. For the small strain regime, the inelastic dilatation is negligible. For higher axial strain values, a decrease in Poisson’s ratio under tension and increase in it under compression are observed. The Drucker–Prager-type equivalent stress and the developed flow rule provide a better description of both the transverse and volumetric strains than that of the classical von Mises–Odqvist flow rules.

## 1. Introduction

Polymer materials are widely used in various engineering fields. An important group of these materials are crystalline polymers, which have especially valuable properties because of their morphology. As the crystallinity increases, the strength and stiffness values, as well as the melting point, increase. Polyoxymethylene (POM) belongs to the class of thermoplastics with crystalline structures. POM is widely applied to high-performance components, because it exhibits higher stiffness, higher ultimate strength and better creep resistance than those of other thermoplastics, e.g., polyethylene and polypropylene. The consumer electronics industry, chemical equipment and mechanical engineering are important areas of application [1]. POM is frequently used in metal–plastic joints; for example, safety-relevant elements for chassis ball joints are made from POM. POM and other thermoplastics are frequently used as matrix materials in composites reinforced by short fibers [2,3,4].

To reduce expensive prototype tests during design or construction optimization, finite element analysis of components is usually performed. The key step in structural analysis is to develop a reliable constitutive model that is able to the reflect basic features of material behavior under multi-axial stress and deformation states. Among many available approaches, the unified constitutive models provide an accurate description of processes such as plasticity, creep, stress relaxation, cyclic hardening/softening and ratcheting. Approaches to develop unified constitutive models are discussed in [5,6,7], among others. The main ingredients include the constitutive equation for the inelastic deformation rate tensor and the evolution equations for internal state variables, for example, the backstress tensor for kinematic hardening.

For polymers, the unified constitutive models were introduced by [8,9] based on the overstress concept. Overstress, also called viscous stress, is the difference between the Cauchy stress tensor and the backstress tensor. The latter is a state variable that enters the constitutive equation for the inelastic deformation rate and is defined by the evolution equation. The overstress-type models were applied and calibrated against experimental data for POM in [10].

Alternatively, rheological models are widely applied to characterize the mechanical behavior of polymeric materials. In [11], a four-element rheological model is proposed to describe primary, secondary and tertiary creep stages of POM considering finite strains. In [12], a six-element rheological model is utilized to describe creep under both static and cyclic loadings.

Although a variety of approaches are available for modeling POM, most of them are usually limited to narrow ranges of stresses, strain rates, temperatures and specific loading paths. For example, the constitutive models presented in [10] are calibrated to room temperature, while in [12], the creep behavior for the fixed temperature of 60 °C is analyzed. The aim of this study is to analyze experimental data on the tensile behavior of POM in a wide range of strain rates and temperatures as well as developing a constitutive model for use in the structural analysis of components. In addition to the previous work, we address the following problems:

- To analyze the behavior of POM, displacement-controlled tensile tests with loading/unloading regimes under different rates and temperatures are performed, and families of stress–strain curves for different strain rates and temperatures are generated.
- Inelastic responses of thermoplastic polymers usually exhibit pressure sensitivity and inelastic dilatation. To analyze inelastic dilatation, digital image correlation (DIC) measurements of transverse strains are performed.
- Polymers exhibit non-linear loading/unloading behavior and strain rate sensitivity, even at room temperature. Although many available constitutive models, for example rheological models, are able to describe non-linearities under constant and monotonic loading, predictions of non-linear unloading responses are usually not accurate. In our study, we apply and develop a composite model of inelastic deformation to characterize the inelastic behavior of POM for both loading and unloading regimes.

To keep the model assumptions transparent, in this study, small strains in the sense of geometrically linear theory are assumed. For the small strain regime, the strains in the uni-axial specimens are uniform over the gage length. Thus, the resulting model can be applied to the analysis of structures subjected to small local strains.

## 2. Basic Features of Material Behavior

In the following section, we discuss the basic features of the material behavior based on our experimental data as well as data from the literature. Strain-controlled tensile tests were performed on standard DIN EN ISO 527 specimens from POM 01-010 (Delrin 500 NC 10${}^{®}$) for temperature levels of −20 °C, 20 °C, 40 °C, 60 °C and 80 °C. For each temperature level, families of stress–strain curves for the four strain rates (0.0001%/s, 0.001%/s, 0.01%/s and 0.1%/s) up to the maximum strain value of 5% were obtained from tests. In addition, unloading after reaching the strain value of 1%, with the same absolute value of the strain rate, was performed. For the selected temperature and strain rate levels, transverse strains were measured for both loading and unloading regimes by applying DIC.

**Figure 1:**
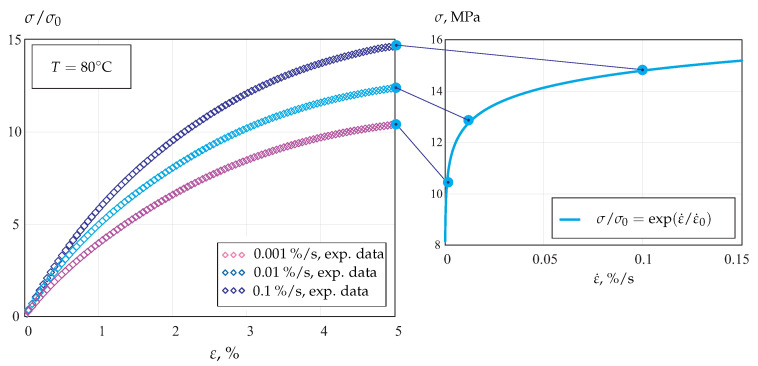
Stress–strain curves for POM at *T* = 80 °C and different strain rates.

Semicrystalline polymers exhibit the significant rate dependence of the tensile behavior. As an example, Figure 1 illustrates stress–strain curves for strain rates of 0.0001%/s, 0.001%/s and 0.01%/s at *T* = 80 °C. The strain rate sensitivity of the flow stress is well approximated by the exponential function of the strain rate, as illustrated in Figure 1.

**Figure 2:**
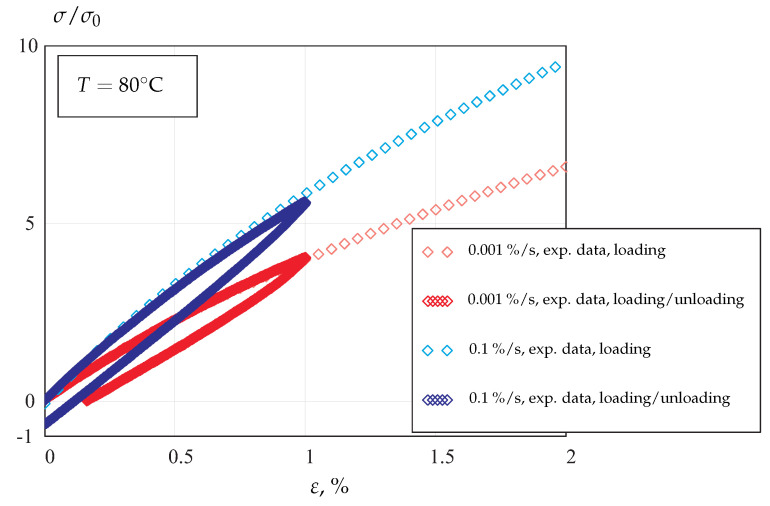
Loading and unloading stress-strain curves for POM at *T* = 80 °C and different strain rates.

In contrast to metals and alloys, semicrystalline polymers usually show non-linear behavior under unloading regimes. Figure 2 illustrates two stress–strain curves obtained at *T* = 80 °C for the constant strain rates of 0.001%/s and 0.1%/s. In addition, loading curves up to the strain value of 1% and subsequent unloading curves under the same rates are presented. Significant non-linearity of the unloading regimes, even for the small strains, are observed. Non-linearity of the unloading curves is enhanced as the maximum strain increases [10]. Furthermore, the shape of the unloading curves significantly depends on the rate of unloading. Stress–strain curves for POM, obtained for the same loading rate but different unloading rates, are presented in [10].

In contrast to metals, the inelastic behavior of polymers is pressure sensitive. Furthermore, change in volume within the inelastic range is usually observed. As shown in [13], for POM at room temperature, the dilatation attains considerable values at moderate and large axial strains (for the strain values higher than 5%). Furthermore, the Poisson ratio usually decreases with an increase in the axial strain under tension. For several semicrystalline polymers, experimental data on transverse strains are presented in [14]. For POM, the decrease in the Poisson ratio was observed in creep tests. The results of measurements applying DIC are discussed in [12].

## 3. Constitutive Model

### 3.1. Composite Model of Inelastic Deformation

**Figure 3:**
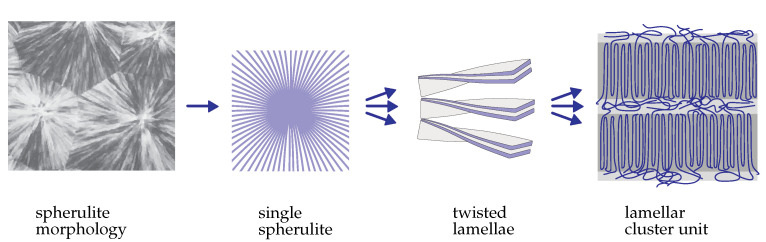
Morphology of crystalline polymers from [15].

For the basic features of the morphology in crystalline polymers, we refer the reader to [15]. Figure 3 provides a sketch of the spherulite microstructure and the composition of individual spherulites.

As many micro-mechanical simulations show, the stress and deformation states inside the spherulites are highly heterogeneous. Indeed, due to the specific structure, the central zone of the spherulite is more stiff than the remaining part is. After the loading, stress concentration and localized inelastic flow in the center of spherulite are usually observed [16,17]. Furthermore, twisted lamellae (Figure 3) are composed of cluster units including amorphous and crystalline zones with significantly different (elastic and inelastic) material properties. Micro-mechanical analyses of representative volumes with an idealized spherulitic microstructure and specific boundary conditions are presented in [16,17,18], among others. The results illustrate that inelastic deformation is highly heterogeneous with different levels of inelastic strains in different microstructural zones. In order to perform a micro-mechanical analysis, constitutive models for amorphous and crystalline zones, as well as non-local cohesive models for their interactions, must be specified. Although the properties of amorphous and crystalline constituents are well known [18,19], the non-local interaction rules remain under discussion. Furthermore, micro-mechanical models are computationally expensive and, therefore, not suitable for the structural analysis of components.

An alternative approach is to develop a composite model (also known as a generalized rheological model or phase mixture model). The basic idea is to approximate the heterogeneous material as a mixture with two or more components (constituents or phases) with different material properties. For stress tensors of each component, phenomenological constitutive equations are formulated. Applying the mixture and interaction rules, the constitutive model of the macroscopic material behavior is developed. The material properties on the microscale, for example, properties of amorphous and crystalline zones, are not required. Instead, basic macroscopic tests, e.g., tension, compression and torsion, as well as tests with loading/unloading regimes, must be performed to generate the experimental data and to identify constitutive functions and material parameters.

The composite model was originally proposed in [20] to describe elastic, plastic and creep deformation in a unified manner. Recently, composite models are widely used to capture the inelastic behavior of many materials under complex loading paths. Examples of metals and alloys are presented in [21,22,23,24,25], among others. Phase mixture models for polymers are applied, for example, in [26,27,28], where amorphous and crystalline phases are considered.

**Figure 4:**
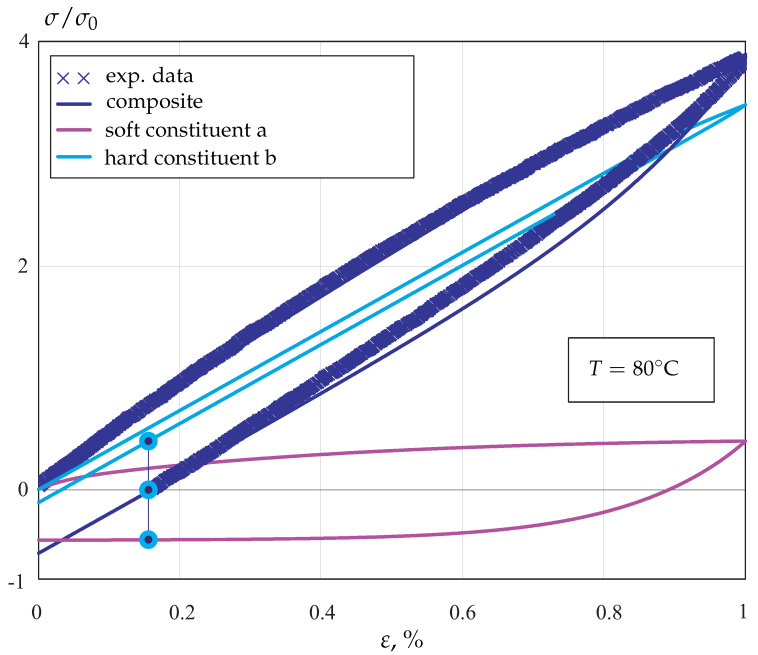
Stress responses under strain-controlled loading and unloading regimes.

To explain the basic idea, let us approximate the heterogeneous deformation of POM by two constituents. We refer to the constituents as a and b. The constituent a is “soft” and applied to model soft regions of the material, including lamellae near the boundaries of spherulite and the amorphous zones. The “hard” constituent is used to characterize regions close to central parts of spherulite, with higher stiffness and higher resistance against inelastic flow. Figure 4 qualitatively illustrates the stress responses of hard and soft constituents during strain-controlled loading and unloading regimes.

In the considered example, the hard constituent behaves nearly linearly, and the elastic has a high Young’s modulus and high flow stress. In contrast, the soft constituent exhibits non-linear behavior after the beginning of loading and has much lower values of Young’s modulus and flow stress. Applying the mixture rule, the complex non-linear loading/unloading profile of the real material can be approximated by the composite model, as shown in Figure 4. After removing the load in the inelastic regime, residual stresses remain in constituents. The corresponding values are designated in Figure 4 by points (tensile stress in the constituent a and compressive stress in the constituent b). These internal stresses determine the subsequent material response; for example, if the material is reloaded, they are responsible for many effects of inelastic behavior, such as the Bauschinger effect, ratcheting and creep recovery. The volume fractions of constituents can be assumed to be dependent on time and/or on the inelastic deformation in order to characterize changes in the material’s microstructure. By changing the variables, the constitutive equation with the backstress tensor can be derived from the composite model, e.g., [22,23]. Therefore, the composite approach provides the generalization of the overstress concept, which is widely used for modeling mechanical responses of polymers.

Below, we approximate the behavior of POM using the composite model with two components. For the sake of simplicity and with regard to the small strains, the volume fraction of constituents is assumed constant.

### 3.2. Constitutive and Evolution Equations

For the stress tensor σ of the composite, the following mixture rule is applied:

(1) $$\sigma =\eta_{a}T_{a}+\left(1-\eta_{a}\right)T_{b}=\sigma_{a}+\sigma_{b},$$

where $T_{a}$ and $T_{b}$ are effective stress tensors of constituents, $\sigma_{a}$ and $\sigma_{b}$ are partial stress tensors, and $\eta_{a}$ is the volume fraction of the soft constituent.

For the deformation rate tensor $D_{k}$, k=a,b, the following additive decompositions in the elastic $D_{k}^{\mathrm{el}}$ and inelastic $D_{k}^{\mathrm{in}}$ parts are applied:

(2) $$D_{k}=D_{k}^{\mathrm{el}}+D_{k}^{\mathrm{in}}$$

For small strains and rotations, the rates of the deformation tensors are the time derivatives of linearized strain tensors $\varepsilon_{k}$ such that

(3) $$D_{k}={\dot{\varepsilon }}_{k},\;\varepsilon_{k}=\varepsilon_{k}^{\mathrm{el}}+\varepsilon_{k}^{\mathrm{in}}$$

For the strain tensors, the following iso-strain rule is assumed:

(4) $$\varepsilon_{a}=\varepsilon_{b}$$

For the stress tensors, the following generalized Hooke’s law is utilized:

(5) $$\sigma_{k}=\lambda_{k}\mathrm{tr}\;\varepsilon_{k}^{\mathrm{el}}I+2\mu_{k}\varepsilon_{k}^{\mathrm{el}},\;k=a,b,$$

where $\lambda_{k}$ and $\mu_{k}$ are Lamé’s constants of *k*-th constituent and I is the second-rank unit tensor (basic rules of the direct tensor calculus are given in [7,29,30], among others). Taking the time derivatives of Equation (5) and applying Equation (2), we obtain the following equations for the stress rates in constituents:

(6) $${\dot{\sigma }}_{k}=\lambda_{k}\mathrm{tr}\;\left(D_{k}-D_{k}^{\mathrm{in}}\right)I+2\mu_{k}\left(D_{k}-D_{k}^{\mathrm{in}}\right)$$

In order to derive the constitutive equations for the inelastic parts of the deformation rate tensors, we follow the flow rule as proposed by [31,32]

(7) $$D_{k}^{\mathrm{in}}=\frac{\partial W_{k}}{\partial \sigma_{k}},$$

where the scalar valued function $W_{k}\left(\sigma_{k}\right)$ plays the role of the inelastic potential. In order to specify the potential, the equivalent stress $\sigma_{{\mathrm{eq}}_{k}}\left(\sigma \right)$ is introduced. Taking into account that $W_{k}\left(\sigma_{k}\right)=W\left(\sigma_{{\mathrm{eq}}_{k}}\left(\sigma_{k}\right)\right)$, the flow rule (7) can be formulated as follows:

(8) $$D_{k}^{\mathrm{in}}={\dot{\varepsilon }}_{k}^{\mathrm{in}}=\frac{\partial W_{k}}{\partial \sigma_{{\mathrm{eq}}_{k}}}\frac{\partial \sigma_{{\mathrm{eq}}_{k}}}{\partial \sigma_{k}}={\dot{\varepsilon }}_{{\mathrm{eq}}_{k}}\frac{\partial \sigma_{{\mathrm{eq}}_{k}}}{\partial \sigma_{k}},\;{\dot{\varepsilon }}_{{\mathrm{eq}}_{k}}=\frac{\partial W_{k}}{\partial \sigma_{{\mathrm{eq}}_{k}}}$$

For isotropic materials, the equivalent stress is a function of three invariants of the stress tensor. Examples of equivalent stress formulations for various materials are discussed in [7,33], among others. In this study, we apply the following expression for the equivalent stress::

(9) $$\sigma_{{\mathrm{eq}}_{k}}=\left\{\begin{array}{cc} \sigma_{{\mathrm{vM}}_{k}}\phi_{k}\left(\frac{\sigma_{m_{k}}}{\sigma_{{\mathrm{vM}}_{k}}}\right) & \sigma_{{\mathrm{vM}}_{k}}>0\\ 0 & \sigma_{{\mathrm{vM}}_{k}}=0 \end{array}\right.,$$

where $\sigma_{m_{k}}=\frac{1}{3}\mathrm{tr}\;\sigma$ is the mean (hydrostatic) stress,

$$\sigma_{{\mathrm{vM}}_{k}}^{2}=\frac{3}{2}\mathrm{tr}\;s_{k}^{2}$$

is the von Mises equivalent stress, and $s_{k}=\sigma_{k}-\sigma_{m_{k}}I$ is the stress deviator. The stress state function $\phi_{k}\left(x\right)$ is defined as follows:

$$\phi_{k}\left(x\right)=\frac{\Omega_{k}\left(x\right)+\left|\Omega_{k}\left(x\right)\right|}{2},\;\Omega_{k}\left(x\right)=\left(1-\xi_{k}\right)+3\xi_{k}x,\;x=\frac{\sigma_{m_{k}}}{\sigma_{{\mathrm{vM}}_{k}}}$$

The weighting factor $\xi_{k}$, $0\leq \xi_{k}\leq 1$ is introduced to consider pressure sensitivity. For $\sigma_{{\mathrm{vM}}_{k}}\neq 0$ and $\Omega_{k}>$, the equivalent stress (9) is a linear combination of the von Mises equivalent stress and the hydrostatic stress as proposed by [34]. The Drucker–Prager-type yield criterion is applied in [13] for modeling the inelastic behavior of POM. With the equivalent stress (9), the flow rule (8) provides the following constitutive equation:

(10) $${\dot{\varepsilon }}_{k}^{\mathrm{in}}={\dot{\varepsilon }}_{{\mathrm{eq}}_{k}}\frac{1+\mathrm{sgn}\Omega_{k}}{2}\left[\left(1-\xi_{k}\right)\frac{3}{2}\frac{s_{k}}{\sigma_{{\mathrm{vM}}_{k}}}+\xi_{k}I\right],\;k=a,b,$$

For $\xi_{k}=0$, the classical von Mises–Odqvist flow rule, valid for inelastically incompressible materials, follows from Equation (10).

For the equivalent inelastic strain rate, the following Prandtl–Eyring law is applied:

(11) $${\dot{\varepsilon }}_{{\mathrm{eq}}_{k}}\left(\sigma_{{\mathrm{eq}}_{k}},H_{k},T\right)=d_{0_{k}}\left(T\right)\sinh\left(\frac{\sigma_{{\mathrm{eq}}_{k}}}{\sigma_{0_{k}}H_{k}}\right),$$

where $d_{0_{k}}\left(T\right)$ is a function of the temperature and $\sigma_{0_{k}}$ is the material parameter to be identified from experimental data. The hardening internal state variable $H_{k}$ is introduced to consider the resistance against inelastic flow. The corresponding evolution equation is assumed as follows:

(12) $${\dot{H}}_{k}=C_{H_{k}}\left(H_{\infty_{k}}-H_{k}\right){\dot{\varepsilon }}_{{\mathrm{eq}}_{k}},\;H_{k}\left(0\right)=1$$

where $C_{H_{k}}$ and $H_{\infty_{k}}$ are material parameters.

With Equations (1)–(5), the following constitutive model for the composite is derived

(13) $$\sigma =\sigma_{m}I+s,\;\sigma_{m}=K\left(\varepsilon_{V}-\varepsilon_{V}^{\mathrm{in}}\right),\;s=2G\left(\epsilon -\epsilon^{\mathrm{in}}\right),$$

where $K=K_{a}+K_{b}$ is the bulk modulus and $G=G_{a}+G_{b}$ is the shear modulus. With Young’s modulus $E_{k}$ and Poisson’s ratio $\nu_{k}$, the bulk and shear moduli are computed as follows:

(14) $$K_{k}=\frac{E_{k}}{3(1-2\nu_{k})},\;G_{k}=\frac{E_{k}}{2(1+\nu_{k})}$$

$\varepsilon_{V}$ is the volumetric strain, and ϵ is the deviatoric part of the strain tensor defined as follows:

$$\varepsilon_{V}=\mathrm{tr}\;\varepsilon ,\;\epsilon =\varepsilon -\frac{1}{3}\varepsilon_{V}I$$

The inelastic parts of the volumetric strain and of the strain deviator are computed as follows:

(15) $$\varepsilon_{V}^{\mathrm{in}}=\left(1-\delta_{K}\right)\varepsilon_{V_{a}}^{\mathrm{in}}+\delta_{K}\varepsilon_{V_{b}}^{\mathrm{in}},\;\epsilon^{\mathrm{in}}=\left(1-\delta_{G}\right)\epsilon_{a}^{\mathrm{in}}+\delta_{G}\epsilon_{b}^{\mathrm{in}},$$

where

(16) $$\delta_{K}=\frac{K_{b}}{K},\;1-\delta_{K}=\frac{K_{a}}{K},\;\delta_{G}=\frac{G_{b}}{G},\;1-\delta_{G}=\frac{G_{a}}{G},$$

and

(17) $$\begin{array}{c} {\dot{\varepsilon }}_{V_{k}}^{\mathrm{in}}=\frac{1+\mathrm{sgn}\Omega_{k}}{2}3\xi_{k}{\dot{\varepsilon }}_{{\mathrm{eq}}_{k}}\left(\sigma_{{\mathrm{eq}}_{k}},H_{k},T\right),\\ {\dot{\epsilon }}_{k}^{\mathrm{in}}=\frac{1+\mathrm{sgn}\Omega_{k}}{2}\frac{3}{2}\left(1-\xi_{k}\right){\dot{\varepsilon }}_{{\mathrm{eq}}_{k}}\left(\sigma_{{\mathrm{eq}}_{k}},H_{k},T\right)\frac{s_{k}}{\sigma_{{\mathrm{vM}}_{k}}},\;k=a,b \end{array}$$

### 3.3. Model Reduction

For the application of the composite model, it is reasonable to reduce the number of material properties to be determined from experimental data. In our analysis, let us assume Poisson’s ratios of the components to be the same, $\nu_{a}=\nu_{b}=\nu$. In this case $\delta_{K}=\delta_{G}=\delta_{E}$, where

$$\delta_{E}=\frac{E_{b}}{E},\;E=E_{a}+E_{b}$$

With Equations (13) and (15), the tensor of inelastic strains can be introduced as follows:

(18) $$\varepsilon^{\mathrm{in}}=\left(1-\delta_{E}\right)\varepsilon_{a}^{\mathrm{in}}+\delta_{E}\varepsilon_{b}^{\mathrm{in}}$$

For the weighting factors $\xi_{k}$, we assume

$$\xi_{a}=\xi_{b}=\xi$$

This assumption leads to the same pressure sensitivity and inelastic dilatation in the components. Furthermore, from our analysis of experimental data, it follows that the hardening saturation parameters $H_{\infty_{k}}$ can be assumed to be the same

$$H_{\infty_{a}}=H_{\infty_{b}}=H_{\infty }$$

The temperature dependencies of the inelastic behavior in Equation (11) are specified as follows:

(19) $$d_{0_{k}}\left(T\right)={\dot{\varepsilon }}_{0_{k}}R\left(T\right),$$

where ${\dot{\varepsilon }}_{0_{k}}$ are material parameters, and R(T) is a function of temperature.

## 4. Model Calibration

### 4.1. Uni-Axial Stress State

In order to calibrate the model, let us consider the following uni-axial stress state:

(20) σ=σe⊗e,

where σ is the uni-axial stress and the unit vector e designates the direction of loading. The partial stress tensors are computed as follows:

(21) $$\sigma_{k}=\sigma_{k}e⊗e+\sigma_{k_{T}}\left(I-e⊗e\right),\;k=a,b,$$

where $\sigma_{k}$ are axial normal stresses and $\sigma_{k_{T}}$ are transverse normal stresses in constituents. From Equations (20) and (21), the following relations can be derived:

(22) $$\sigma =\sigma_{a}+\sigma_{b},\;\sigma_{a_{T}}+\sigma_{b_{T}}=0$$

The values of transverse normal stresses $\sigma_{k_{T}}$ are usually much lower than the axial stress values and can be neglected in the first step of model calibration (the finite element simulations of the tensile test show that the absolute values of transverse stresses are less than 0.02|σ|). The strain states are characterized by the following tensors:

(23) $$\varepsilon =\varepsilon e⊗e+\varepsilon_{T}\left(I-e⊗e\right),\;\varepsilon_{k}=\varepsilon_{k}e⊗e+\varepsilon_{k_{T}}\left(I-e⊗e\right),\;k=a,b,$$

where ε and $\varepsilon_{k}$ are the longitudinal strains and $\varepsilon_{T}$ and $\varepsilon_{k_{T}}$ are the transverse strains. For the stress and strain states defined by Equations (20), (21) and (23), the constitutive Equations (1)–(12) take the following form

(24) $$\begin{array}{c} \sigma_{k}=E_{k}\left(\varepsilon -\varepsilon_{k}^{\mathrm{in}}\right),\;k=a,b,\\ {\dot{\varepsilon }}_{k}^{\mathrm{in}}=\frac{1+\mathrm{sgn}\Omega_{k}}{2}d_{0_{k}}\left(T\right)\sinh\left(\frac{\sigma_{{\mathrm{eq}}_{k}}}{\sigma_{0_{k}}H_{k}}\right)\left[\left(1-\xi \right)\mathrm{sgn}\left(\sigma_{k}\right)+\xi \right],\\ {\dot{H}}_{k}=C_{H_{k}}\left(H_{\infty }-H_{k}\right)\left|{\dot{\varepsilon }}_{k}^{\mathrm{in}}\right|,\\ \sigma_{{\mathrm{eq}}_{k}}=\left|\sigma_{k}\right|\frac{\Omega_{k}+\left|\Omega_{k}\right|}{2},\;\Omega_{k}=\left(1-\xi \right)+\xi \mathrm{sgn}\left(\sigma_{k}\right) \end{array}$$

With Equations (20)–(24), the stress and the inelastic strain of composite are computed as follows:

(25) $$\sigma =E\left(\varepsilon -\varepsilon^{\mathrm{in}}\right),\;\varepsilon^{\mathrm{in}}=\left(1-\delta_{E}\right)\varepsilon_{a}^{\mathrm{in}}+\delta_{E}\varepsilon_{b}^{\mathrm{in}}$$

For any given strain-controlled loading path ε(t), where *t* is the time variable, the constitutive Equation (25) and the evolution Equation (24)${}_{2}$ and (24)${}_{3}$ can be integrated with respect to the time providing the stress response σ(t). With the available experimental data for σ(t), the material parameters and the function of temperature *R* can be identified.

For the loading in the tensile regime, we set $\sigma_{k}>0$. Equations (24)${}_{2}$ and (24)${}_{3}$ take the following form

(26) $$\begin{array}{c} {\dot{\varepsilon }}_{k}^{\mathrm{in}}=d_{0_{k}}\left(T\right)\sinh\left(\frac{\sigma_{k}}{\sigma_{0_{k}}H_{k}}\right),\\ {\dot{H}}_{k}=C_{H_{k}}\left(H_{\infty }-H_{k}\right){\dot{\varepsilon }}_{k}^{\mathrm{in}} \end{array}$$

Let us assume that a steady-state flow regime exists, such that ${\dot{\sigma }}_{k}=0$, $\dot{\sigma }=0$, ${\dot{H}}_{k}=0$. In this case, the stresses and the hardening variables attain the asymptotic values $\sigma_{k}=\sigma_{f_{k}}$, $\sigma =\sigma_{f}$ and $H_{k}=H_{\infty }$, where $\sigma_{f_{k}}$ and $\sigma_{f}$ are flow stresses. Setting the rates of the stresses to zero, Equations (24)${}_{1}$ and (25) yield

$${\dot{\varepsilon }}_{k}^{\mathrm{in}}={\dot{\varepsilon }}^{\mathrm{in}}=\dot{\varepsilon }$$

From Equation (26)${}_{1}$, the following relationships between the strain rate and the flow stresses can be obtained:

(27) $$\dot{\varepsilon }=d_{0_{k}}\left(T\right)\sinh\left(\frac{\sigma_{f_{k}}}{\sigma_{0_{k}}H_{\infty }}\right)$$

### 4.2. Identification Procedure

In order to identify the material parameters, the following step-by-step procedure is applied

1. Smooth experimental data and compute stress rates;
2. Identify the Young’s modulus as a function of temperature;
3. Compute inelastic strains and strain rates for each temperature and strain rate level;
4. Identify flow stresses as functions of strain rate and temperature;
5. Identify parameters in the composite model from families of stress–strain curves for different strain rates and temperature levels;
6. Identify Poisson’s ratios (elastic and inelastic) and the parameter ξ from transverse strains, measured by DIC.

**Figure 5:**
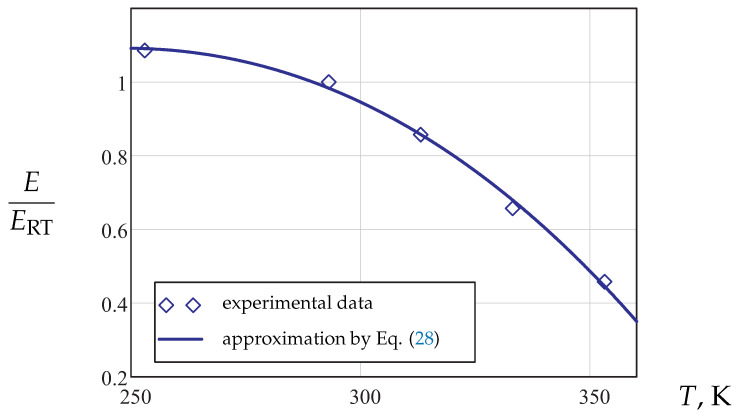
Normalized Young’s modulus vs. temperature.

Figure 5 illustrates the normalized Young’s modulus as a function of the absolute temperature, where $E_{\mathrm{RT}}$ is the Young’s modulus at room temperature.

This dependence is approximated by the following relation:

(28) $$E\left(T\right)=A_{E}+B_{E}T+C_{E}T^{3},$$

where $A_{E}$, $B_{E}$ and $C_{E}$ are material parameters.

In a steady-state flow regime, the hyperbolic sine functions in Equation (27) can be approximated by the exponents as follows:

(29) $$\frac{\dot{\varepsilon }}{{\dot{\varepsilon }}_{0_{k}}R\left(T\right)}=\frac{1}{2}\exp\left(\frac{\sigma_{f_{k}}}{\sigma_{0_{k}}H_{\infty_{k}}}\right)$$

with

$$\sigma_{f}=\sigma_{f_{a}}+\sigma_{f_{b}}$$

and Equation (29), the following relationship between the strain rate and flow stress can be derived:

(30) $$\frac{\dot{\varepsilon }}{{\dot{\varepsilon }}_{0}R\left(T\right)}=\frac{1}{2}\exp\left(\frac{\sigma_{f}}{\sigma_{0}}\right),$$

where

$$\sigma_{0}=\sigma_{0_{a}}H_{\infty_{a}}+\sigma_{0_{b}}H_{\infty_{b}},\;\sigma_{0}\ln{\dot{\varepsilon }}_{0}=\sigma_{0_{a}}H_{\infty_{a}}\ln{\dot{\varepsilon }}_{0_{a}}+\sigma_{0_{b}}H_{\infty_{b}}\ln{\dot{\varepsilon }}_{0_{b}}$$

**Figure 6:**
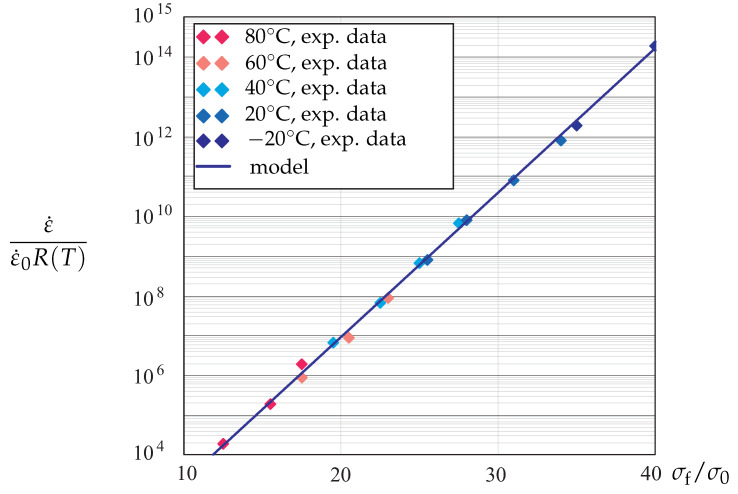
Normalized strain rate vs. flow stress.

Figure 6 shows the strain rate as a function of flow stress. To normalize the strain rates, the following generalized Arrhenius functions of temperature is applied:

(31) $$R\left(T\right)=\exp\left(-\frac{\alpha }{T}\right)\left\{1+\exp\left[-\left(\alpha -\alpha_{L}\right)\left(\frac{1}{T_{*}}-\frac{1}{T}\right)\right]\right\},$$

where α, $\alpha_{L}$ and $T_{*}$ are material parameters.

We observe that the exponential function of stress (30) and the generalized Arrhenius functions of temperature (31) approximate the strain rate sensitivity for a wide range of temperatures with satisfactory accuracy.

**Figure 7:**
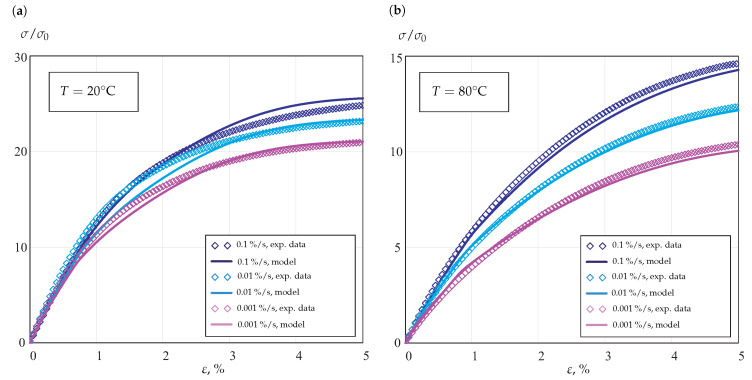
Stress–strain curves under monotonic loading with different strain rates: (**a**) 20 °C; (**b**) 80 °C.

To identify the material parameters in the composite model, the stress–strain curves under loading and unloading regimes for each strain rate and temperature were applied. As an example, Figure 7 shows the experimental data and the results of calibration for stress responses under monotonic loading with different strain rates and temperature levels of 20 °C and 80 °C.

**Figure 8:**
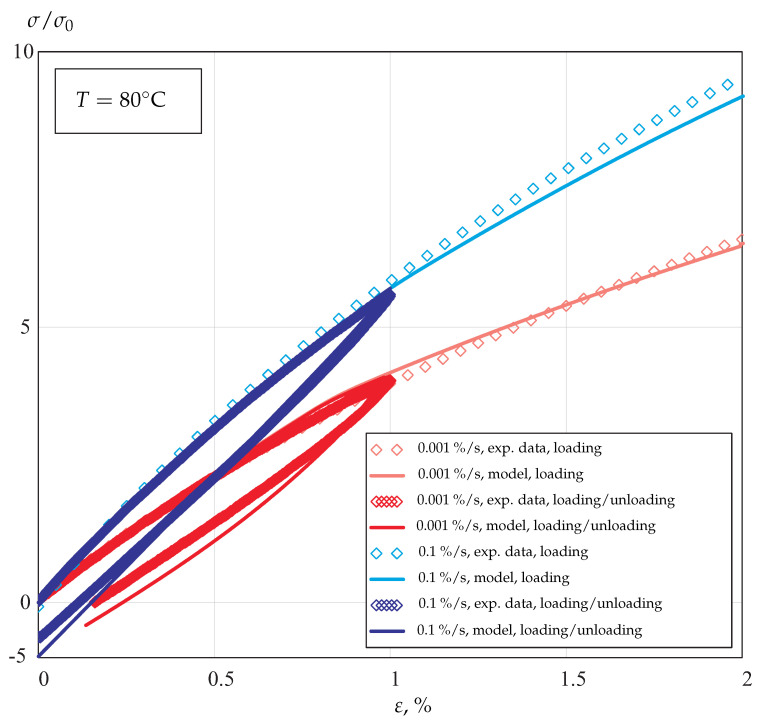
Stress-strain curves under monotonic loading and unloading with different strain rates at 80 °C.

The results of calibration for loading and unloading regimes are presented in Figure 8.

The results show that the developed composite model with the introduced constitutive functions of stress and temperature is able to predict the tensile behavior in a wide range of strain rates and temperatures. Table 1 and Table 2 provide summaries on the values of the identified material parameters.

### 4.3. Transverse Strain and Inelastic Dilatation

**Figure 9:**
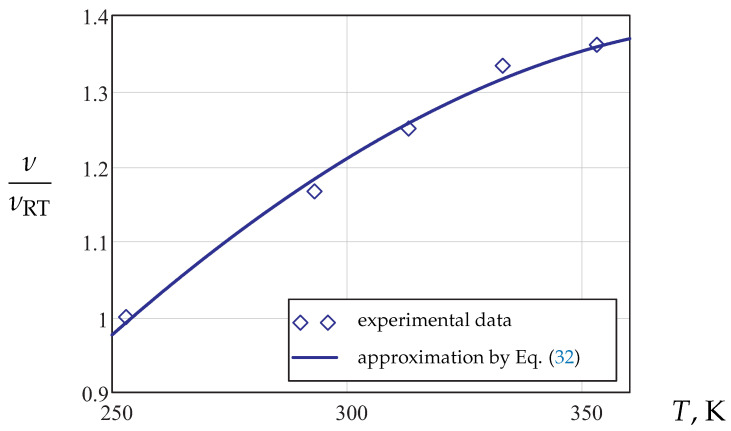
Normalized Poisson’s ratio vs. temperature.

Based on the results of the transverse strain measurements from DIC within the range of small longitudinal strains less than 1%, the following approximation of the Poisson’s ratio was established:

(32) $$\nu \left(T\right)=A_{\nu }+B_{\nu }T+C_{\nu }T^{3},$$

where $A_{\nu }$, $B_{\nu }$ and $C_{\nu }$ are material parameters. Figure 9 illustrates the normalized Poisson’s ratio as a function of the absolute temperature, where $\nu_{\mathrm{RT}}$ is the corresponding value at room temperature. For the considered temperature range, Equation (32) is applicable to approximate the data with satisfactory accuracy.

The transverse strains of the constituent $\varepsilon_{T_{k}}$ and the transverse strain of composite $\varepsilon_{T}$ can be computed as follows:

(33) $$\varepsilon_{T}=\varepsilon_{T_{k}}^{\mathrm{el}}+\varepsilon_{T_{k}}^{\mathrm{in}}=-\frac{\nu }{E}\sigma +\varepsilon_{T_{k}}^{\mathrm{in}},\;\varepsilon_{T}=\varepsilon_{T}^{\mathrm{el}}+\varepsilon_{T}^{\mathrm{in}}=-\frac{\nu }{E}\sigma +\varepsilon_{T}^{\mathrm{in}},$$

Let us introduce the inelastic Poisson’s ratios as follows:

(34) $$\nu_{k}^{\mathrm{in}}=-\frac{\varepsilon_{T_{k}}^{\mathrm{in}}}{\varepsilon_{k}^{\mathrm{in}}}$$

With Equations (18) and (34), the transverse strain of the composite can be related to the longitudinal strains of constituents as follows:

(35) $$\varepsilon_{T}^{\mathrm{in}}=-\left(1-\delta_{E}\right)\nu_{a}^{\mathrm{in}}\varepsilon_{a}^{\mathrm{in}}-\delta_{E}\nu_{b}^{\mathrm{in}}\varepsilon_{b}^{\mathrm{in}}$$

From the constitutive Equation (10), we obtain

(36) $$\nu_{k}^{\mathrm{in}}=-\frac{\xi -0.5\left(1-\xi \right)\mathrm{sgn}\sigma_{k}}{\xi +\left(1-\xi \right)\mathrm{sgn}\sigma_{k}},$$

For monotonic loading (tensile or compressive), the stresses in constituents have the same sign and $\mathrm{sgn}\sigma_{k}=\mathrm{sgn}\sigma$. In this case, Equation (36) is simplified as follows:

(37) $$\nu_{a}^{\mathrm{in}}=\nu_{b}^{\mathrm{in}}=-\frac{\xi -0.5(1-\xi )\mathrm{sgn}\sigma }{\xi +(1-\xi )\mathrm{sgn}\sigma },$$

With $\nu_{a}^{\mathrm{in}}=\nu_{b}^{\mathrm{in}}=\nu^{\mathrm{in}}$, Equation (35) yields

(38) $$\varepsilon_{T}^{\mathrm{in}}=-\nu^{\mathrm{in}}\left[\left(1-\delta_{E}\right)\varepsilon_{a}^{\mathrm{in}}+\delta_{E}\varepsilon_{b}^{\mathrm{in}}\right]=-\nu^{\mathrm{in}}\varepsilon^{\mathrm{in}}$$

From Equations (33), (37) and (38), the transverse strain is computed as follows:

(39) $$\varepsilon_{T}=-\nu \frac{\sigma }{E}-\nu^{\mathrm{in}}\left(\varepsilon -\frac{\sigma }{E}\right),\;\nu^{\mathrm{in}}=-\frac{\xi -0.5(1-\xi )\mathrm{sgn}\sigma }{\xi +(1-\xi )\mathrm{sgn}\sigma }$$

For the volumetric strain $\varepsilon_{V}=\varepsilon +2\varepsilon_{T}$, we obtain

(40) $$\varepsilon_{V}=\left(1-2\nu \right)\frac{\sigma }{E}+\left(1-2\nu^{\mathrm{in}}\right)\left(\varepsilon -\frac{\sigma }{E}\right)$$

The inelastic Poisson’s ratio $\nu^{\mathrm{in}}$ is not a material property since it depends on the kind of stress state. It is related to the weighting factor ξ according to Equation (39)${}_{2}$. This relation follows according to the assumed equivalent stress (9) and the flow rule (10). In the case of tension, we have

(41) $$\nu^{\mathrm{in}}=\frac{1}{2}\left(1-3\xi \right),$$

while for the uni-axial compression

(42) $$\nu^{\mathrm{in}}=\frac{1}{2}\frac{1+\xi }{1-2\xi }$$

Many materials, for example, metals and alloys, exhibit negligible change in volume in the course of inelastic deformation. With ξ=0, Equations (17)${}_{1}$ and (15) yield ${\dot{\varepsilon }}_{V_{k}}^{\mathrm{in}}=0$ and ${\dot{\varepsilon }}_{V}^{\mathrm{in}}=0$. Furthermore, Equation (39)${}_{2}$ provides $\nu^{\mathrm{in}}=0.5$ independently on the kind of stress state.

**Figure 10:**
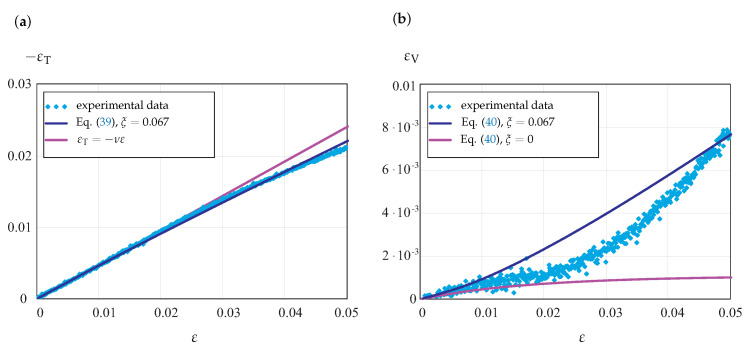
Experimental data for POM from tension tests at 40 °C and DIC measurements. (**a**) Transverse strain vs. axial strain; (**b**) volumetric strain vs. axial strain.

Several tension tests with DIC measurements of strains were performed under selected temperature and strain rate levels for longitudinal strains up to 5%. As an example, the results for *T* = 40 °C and $\dot{\varepsilon }=0.1$%/s are presented in Figure 10.

Figure 10a illustrates the transverse strain vs. longitudinal strain. For the comparison, the straight line corresponding to the linear elastic behavior with $\varepsilon_{T}=-\nu \varepsilon$ is presented. With an increase in loading, the actual curve deviates from the linear elastic regime such that $|\varepsilon_{T}|<\nu \varepsilon$. Therefore, with an increase in inelastic deformation, the actual Poisson’s ratio decreases. The decrease in Poisson’s ratio is documented in [14] for several semicrystalline polymers. Applying Equation (39) with ξ=0.067, the transverse strain vs. longitudinal strain can be described with satisfactory accuracy, as shown in Figure 10a.

From the results of DIC measurements, the change in volume of the uni-axial specimen can be approximately evaluated. Figure 10b illustrates the volumetric strain, computed by the formula $\varepsilon_{V}=\varepsilon +2\varepsilon_{T}$, as a function of the longitudinal strain. For comparison, the plots of Equation (40) for $\nu^{\mathrm{in}}=0.4$, ξ=0.067 and $\nu^{\mathrm{in}}=0.5$ξ=0 are presented. The assumption of inelastic incompressibility leads to inaccurate results for the change in volume, in particular for axial strains ε>0.02. With the equivalent stress (9) and the flow rule (10), the results are closer to the experimental data. However, the non-linearity of the actual volumetric strain vs. axial strain response is not captured accurately.

**Figure 11:**
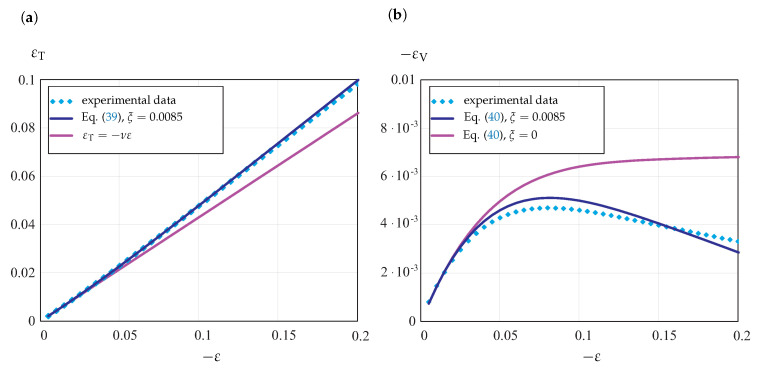
Experimental data for POM from compression tests at 20 °C from [13]. (**a**) Transverse strain vs. axial strain; (**b**) volumetric strain vs. axial strain.

In [10,13], experimental data for POM samples subjected to compression are discussed. Axial stress vs. axial strain curves as well as volumetric strain vs. axial strain curves are presented. Figure 11 illustrates the experimental data for the transverse strain and the volumetric strain.

The results of computations with Equations (39) and (40) are presented. With ξ=0.0085, the predictions by the constitutive model agree well with experimental data. The actual experimental values for the transverse strain lie above the straight line corresponding to the elastic regime, as shown in Figure 11a. This indicates that the Poisson’s ratio increases in the course of inelastic deformation under compression. This feature is well reproduced by Equation (39) with ξ=0.0085. If we assume $\nu^{\mathrm{in}}=0.5$, the volumetric strain approaches the asymptotic steady-state flow value, as shown in Figure 11b. Compared to this, the absolute value of the volumetric strain decreases, as shown by both the experimental data and the prediction of Equation (40).

It should be noted that the parameter ξ is also responsible for the pressure sensitivity and the tension compression asymmetry. For ξ=0, the model provides the same behavior under tension and compression. With the identified low values of ξ, only a weak difference in tensile and compressive curves can be expected for the considered strain range. Therefore, the equivalent stress (9) should be refined in the future. To this end, a systematic experimental analysis of both the volumetric deformation and pressure sensitivity is required.

## 5. Conclusions

The aim of this study was to analyze experimental data for the inelastic response of POM in a wide range of strain rates and at various temperatures as well as developing and calibrating the constitutive model for the material’s behavior. The composite model of inelastic deformation was applied and further developed to capture the non-linearity of the stress responses for both loading and unloading regimes. To consider the transverse strain and the volumetric strain responses, the Drucker–Prager-type equivalent stress formulations were combined with the Odqvist flow rule. The predictions by the constitutive law were compared with experimental data from stress vs. axial strain curves as well as with strain measurements from DIC. Based on the results, we may conclude the following:

- The developed composite model is able to capture the non-linearity of stress–strain curves for loading and unloading paths within the small strain regime (axial strains up to 5%). For higher strains, apart from geometrically non-linear theory, several model assumptions should be refined. In particular, for the volume fraction of the constituents, appropriate evolution laws should be formulated and calibrated.
- The Prandtl–Eyring constitutive function of stress (11) is well applicable to describe the strain rate sensitivity in a wide range, from $10^{-4}$%/s to 0.1%/s.
- To capture the temperature dependence of tensile behavior from −20 °C to 80 °C, the generalized Arrhenius functions of temperature (31) are required.
- For the small strain regime (axial strains up to 1–2%), the inelastic dilatation is small and can be neglected. For higher axial strain values, the decrease in Poisson’s ratio under tension and increase it under compression are observed.
- The Drucker–Prager-type equivalent stress (9) and the flow rule (10) provide a better description of both the transverse and volumetric strains than that of the classical von Mises–Odqvist flow rules. However, for higher values of the axial strain, the non-linearity of the actual volumetric strain vs. axial strain response is not accurately captured. Furthermore, the tension compression asymmetry is underestimated.

Further studies should be related to the systematic experimental research of the material response for moderate and large strains in order to refine the constitutive model:

- Non-linearity of stress responses for loading/unloading paths under different strain rates should be analyzed.
- The applicability of the model to the lower strain rate regimes of creep and stress relaxation should be examined.
- Systematic analysis of experimental data on transverse strains based on DIC measurements for a wide range of axial stains under tension and compression should be performed.

## Figures and Tables

**Table 1:** Material parameters in the composite model.

Component a			Component b		
**Parameter**	**Unit**	**Value**	**Parameter**	**Unit**	**Value**
$E_{a}$	MPa	1536	$E_{b}$	MPa	1907
$d_{0_{a}}$	-	$2.239\cdot 10^{-9}$	$d_{0_{b}}$	-	$1.396\cdot 10^{-22}$
$\sigma_{0_{a}}$	MPa	1.263	$\sigma_{0_{b}}$	MPa	1.131
$C_{H_{a}}$	s	486.823	$C_{H_{b}}$	s	168.462

**Table 2:** Material parameters for temperature dependencies.

Parameter	Unit	Value
$A_{E}$	MPa	$-3.692\cdot 10^{3}$
$B_{E}$	$\frac{\mathrm{MPa}}{K}$	45.316
$C_{E}$	$\frac{\mathrm{MPa}}{K^{3}}$	$-2.442\cdot 10^{-4}$

## Data Availability

The data presented in this study are partly available on request from the corresponding author and coauthors. The data are partly not publicly available due to confidentiality.
